# High-throughput profiling of diapause regulated genes from *Trichogramma dendrolimi* (Hymenoptera: Trichogrammatidae)

**DOI:** 10.1186/s12864-020-07285-4

**Published:** 2020-12-04

**Authors:** Xue Zhang, Wenmei Du, Junjie Zhang, Zhen Zou, Changchun Ruan

**Affiliations:** 1 Engineering Research Center of Natural Enemies, Institute of Biological Control, Jilin Agricultural University, Changchun, 130118 China; 2 State Key Laboratory of Integrated Management of Pest Insect and Rodents, Institute of Zoology, Chinese Academy of Sciences, Beijing, 100101 China

**Keywords:** *Trichogramma dendrolimi*, Transcriptome, RNA-Seq, Diapause, Diapause-related genes

## Abstract

**Background:**

The parasitoid wasp, *Trichogramma dendrolimi*, can enter diapause at the prepupal stage. Thus, diapause is an efficient preservation method during the mass production of *T. dendrolimi*. Previous studies on diapause have mainly focused on ecological characteristics, so the molecular basis of diapause in *T. dendrolimi* is unknown. We compared transcriptomes of diapause and non-diapause *T. dendrolimi* to identify key genes and pathways involved in diapause development.

**Results:**

Transcriptome sequencing was performed on diapause prepupae, pupae after diapause, non-diapause prepupae, and pupae. Analysis yielded a total of 87,022 transcripts with an average length of 1604 bp. By removing redundant sequences and those without significant BLAST hits, a non-redundant dataset was generated, containing 7593 sequences with an average length of 3351 bp. Among them, 5702 genes were differentially expressed. The result of Gene Ontology (GO) enrichment analysis revealed that regulation of transcription, DNA-templated, oxidation-reduction process, and signal transduction were significantly affected. Ten genes were selected for validation using quantitative real-time PCR (qPCR). The changes showed the same trend as between the qPCR and RNA-Seq results. Several genes were identified as involved in diapause, including *ribosomal proteins, zinc finger proteins, homeobox proteins, forkhead box proteins, UDP-glucuronosyltransferase*, *Glutathione-S-transferase*, *p53*, and *DNA damage-regulated gene 1* (*pdrg1*). Genes related to lipid metabolism were also included.

**Conclusions:**

We generated a large amount of transcriptome data from *T. dendrolimi*, providing a resource for future gene function research. The diapause-related genes identified help reveal the molecular mechanisms of diapause, in *T. dendrolimi*, and other insect species.

**Supplementary Information:**

The online version contains supplementary material available at 10.1186/s12864-020-07285-4.

## Background

The insect egg parasitoid, *Trichogramma dendrolimi* Matsumura (Hymenoptera: Trichogrammatidae), is used as a biological control agent against several important Lepidopteran insect pests, such as *Chilo suppressalis* [1], *Cnaphalocrocis medinalis* [2], and *Ostrinia furnacalis* [3]. Application of *T. dendrolimi* is usually involves inundative release into fields, so large numbers of *T. dendrolimi* are needed in biological control programs. Preservation of parasitoids to assure their supply is essential for the continuous production of parasitoids throughout the year [4]. Cold storage of parasitized host eggs is the most commonly used method for parasitoid preservation [5]. Although *Trichogramma* can be kept for a long period at low temperatures, their performance, such as emergence rate and longevity, decreases as storage time increases. For example, the survival rate of *T. dendrolimi* significantly decreases after 3 weeks of storage at low temperatures [6], and similar decreases have been found in other *Trichogramma* species [7, 8]. Therefore, it is important to improve the effectiveness of preservation. Diapause can be an effective mechanism to solve this problem.

Diapause is an essential processes that helps insects avoid periods that are unfavorable for growth and development [9]. Insects can utilize diapause to resist adverse environmental conditions. When future environmental conditions likely become unsuitable, diapause may be initiated to reduce energy and metabolic activity, enhance stress resistance, and extend lifespan [10–12]. Many aspects of diapause have been comprehensively reviewed in insects, and these show that diapause is a complicated process [13–15]. Some studies have focused on optimized conditions for diapause induction or termination to improve biological control programs [16–20]. Several diapause-associated genes, such as *dilp1*, forkhead box protein O (*foxo*), and *akt*, have been identified in insect species [21–26]. Few studies, however, have examined the molecular mechanism of diapause in *Trichogramma* spp. Many phenomena related to diapause in *T. dendrolimi* are unexplained. For example, adults of *T. dendrolimi* that have experienced diapause development produce more eggs [27]. Therefore, it is necessary to better understand the diapause mechanism. Transcriptome sequencing is useful for gene expression research, and many studies have used RNA-sequencing (RNA-Seq) to address a variety of problems. Some studies have focused on insect resistance to insecticides [28–31]. Other studies have examined insect adaptability to extreme environments [32, 33], or focus on selected areas of the genome such as chemosensory genes [34]. There are few studies on insect diapause using RNA-Seq, but Hao et al. (2019) identified the candidate genes (*rai1* and *foxo*) related to the FOXO pathway in the egg diapause regulation of *Locusta migratoria* [35].

The objective of this study was to use RNA-Seq to characterize diapause-related genes in *T. dendrolimi*. We report the gene expression profiles of diapause and non-diapause *T. dendrolimi*. The results of this study are expected to provide a reference for deciphering the diapause mechanism in *T. dendrolimi* and guiding the use of *T. dendrolimi* in biological control programs.

## Results

### Diapause induction, termination, sequencing, and gene identification

After the completion of the diapause induction process, the parasitized host eggs were dissected to verify whether *T. dendrolimi* entered diapause successfully. Diapausing parasitoids remain at the prepupal stage (Dpre). If diapause induction fails, the prepupae would die or continue to develop into pupae and adults. Following the diapause termination process, the parasitized host eggs were transferred to normal development conditions (26 °C ± 1 °C, 60% ± 5% RH, 16:8 h L:D). If diapause was disrupted, the parasitoids would transform from prepupa to pupa within several days, noted as Dp. However, if diapause was not disrupted, the *T. dendrolimi* would remain in the prepupa stage. In this study, 99% of the parasitoids entered diapause successfully, and about 95% resumed development after the 70 d termination treatment. The prepupae and pupae of *T. dendrolimi* that developed under normal conditions were obtained as reference groups noted as NDpre and NDp, respectively.

RNA samples obtained from distinct stages of *T. dendrolim*i were prepared and sequenced using the Illumina Hiseq2000 sequencing platform. Four cDNA libraries were constructed from the samples of Dpre, Dp, NDpre, and NDp. After filtering raw reads (reads containing adaptors, reads containing N larger than 10%, and low-quality reads (Qphred < 20) were removed), clean reads were retained (Table 1). The clean data were assembled by Trinity and Corset with 87,022 transcripts, and an average length of 1604 nt and an N50 of 3148. Of the transcripts, 35,231 (40.5%) were longer than 1000 bp (Table 2).

**Table 1:** Summary of Illumina transcriptome assembly for *T. dendrolimi*

Sample name	Raw reads	Clean reads	Clean bases	Error rate (%)	Q20 (%)	Q30 (%)	GC content (%)
Dpre-A	49,071,974	47,695,622	7.15G	0.03	96.52	91.43	41.30
Dpre-B	49,810,990	47,210,830	7.08G	0.01	97.26	93.17	43.77
Dpre-C	47,347,676	44,732,274	6.71G	0.01	97.29	93.25	43.84
Dp-A	49,517,974	48,189,484	7.23G	0.02	95.98	90.34	41.11
Dp-B	51,270,660	48,977,252	7.35G	0.02	96.97	92.69	40.74
Dp-C	45,943,884	43,816,628	6.57G	0.02	96.84	92.38	42.41
NDpre-A	45,701,770	44,515,808	6.68G	0.01	96.28	90.97	40.07
NDpre-B	50,860,900	48,522,830	7.28G	0.01	97.37	93.48	41.57
NDpre-C	47,589,266	45,544,062	6.83G	0.01	97.36	93.45	38.73
NDp-A	56,029,606	54,463,466	8.17G	0.01	96.88	92.35	38.69
NDp-B	46,605,166	44,410,060	6.66G	0.01	97.19	93.11	40.63
NDp-C	62,287,702	59,900,798	8.99G	0.01	97.24	93.26	40.71

Note: A, B, and C represent the three biological replicates of each sample

**Table 2:** General features of the de novo assembled transcriptome by Trinity

	Transcripts	Unigenes
200–500 bp	131,509	22,869
500–1 kbp	42,525	28,922
1 k-2 kbp	18,308	18,119
> 2 kbp	17,115	17,112
Total	209,457	87,022
Min length	201	201
Mean length	865	1604
Median length	389	814
Max length	29,327	29,327
N50	1732	3148
N90	308	647
Total nucleotides	181,250,941	139,571,319

**Fig. 1:**
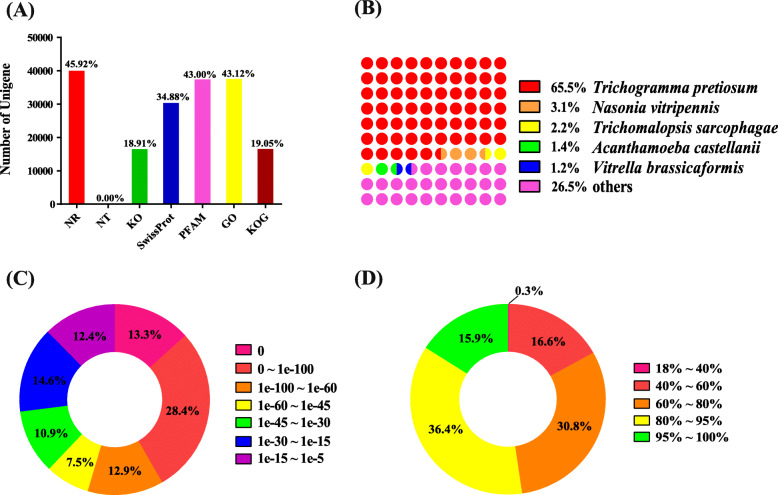
Sequence annotation and homology search against NR database for *T. dendrolimi* unigenes. **a** Annotation results in seven major databases. **b** Distribution of species of top BLAST hits. **c** Distribution of E-values of top BLAST hits with a cut-off E-value of 1e-5. **d** Distribution of similarity of top BLAST hits

To study gene function, transcripts were annotated using BLASTX searches against the non-redundant (NR) sequence database; 39,969 (45.92%) displayed homology to known proteins (E < 1e^− 5^; Fig. 1a). Nearly 25,000 annotated transcripts, over 65% of the annotated transcripts, were homologous to *T. pretiosum*, probably because the genome of *T. pretiosum* was the only available *Trichogramma*. Fewer transcripts were homologous to *Nasonia vitripennis* (1217, 3.1%), *Apis florea* (26, 0.03%), *A. dorsata* (12, 0.01%), or *A. cerana* (15, 0.01%) (Fig. 1b). Fewer than 40 transcripts matched those from *Microplitis demolitor*. Among all annotated transcripts, 73.0% had significant homology with an E-value of < 10^− 30^ (Fig. 1c), and 52.3% had a similarity greater than 80.0% (Fig. 1d). After filtering and removing redundant sequences, we retained those with significant BLAST hits and constructed a non-redundant dataset containing 7593 unigenes with an average length of 3351 nt. Based on the annotation, such as gene length, ID, and speculative function, the diapause-related genes and potential genes involved in diapause were sorted out for further analysis.

### Identification of DEGs and functional classification

**Fig. 2:**
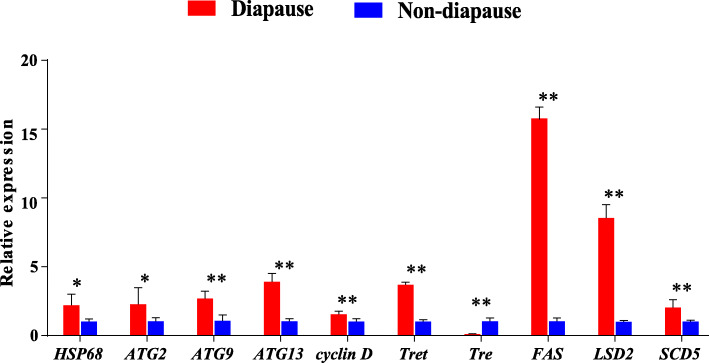
Relative expressions of 10 selected genes analyzed by qPCR analysis. Nine genes were up-regulated, and one gene was down-regulated during the diapause stage. The changing trends of all 10 genes between diapause and non-diapause were identical between qPCR and RNA-Seq. The red bar represents the diapause, while the blue bar represents the non-diapause. The relative mRNA levels are represented as the mean ± S.D. *, *p* < 0.05; **, *p* < 0.01

Ten genes were selected for validation with qPCR, and glyceraldehyde phosphate dehydrogenase (*GAPDH*) was selected as the reference gene after measuring its stable expression level in diapause and non-diapause groups. The tendencies of the expression profiles of these genes were similar according to RNA-Seq and qPCR (Fig. 2). Among these 10 selected genes, all except *trehalase* (*tre*) were up-regulated during diapause.

**Fig. 3:**
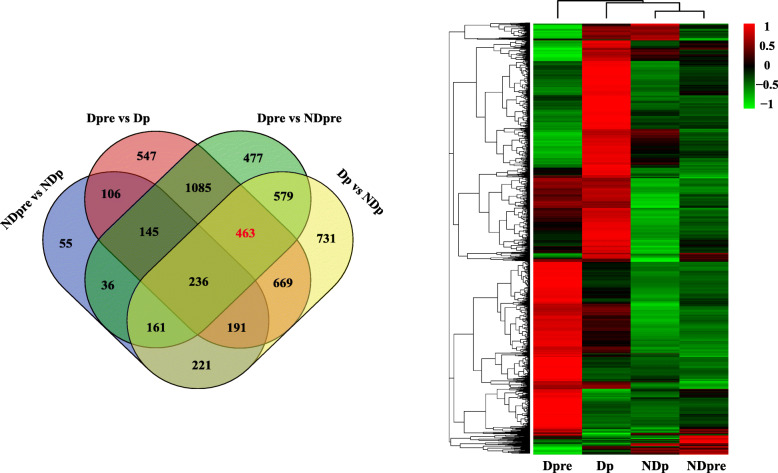
Venn graph and heatmap of DEGs across the four treatments. In the heatmap graph, red indicates relatively high expression, green indicates relatively low expression, and black represents moderate expression

To study diapause-specific transcriptional changes in *T. dendrolimi* induced by low temperature, we made pairwise comparisons between different libraries to identify the DEGs. A total of 5702 DEGs were identified among four groups. Among these DEGs, there were 3182 DEGs changed in Dpre compared to NDpre. DESeq identified 3251 and 3442 DEGs exclusively changed in Dp vs NDp and Dpre vs Dp, respectively. In addition, the DEGs changed in NDpre vs NDp were 1511. This group of DEGs may be the genes related to normal development, namely from prepupa to pupa, not to diapause development. According to the Venn diagram, there were 463 genes changed throughout the diapause development process, while in the normal development process, the expression of these genes did not change (Fig. 3).

**Fig. 4:**
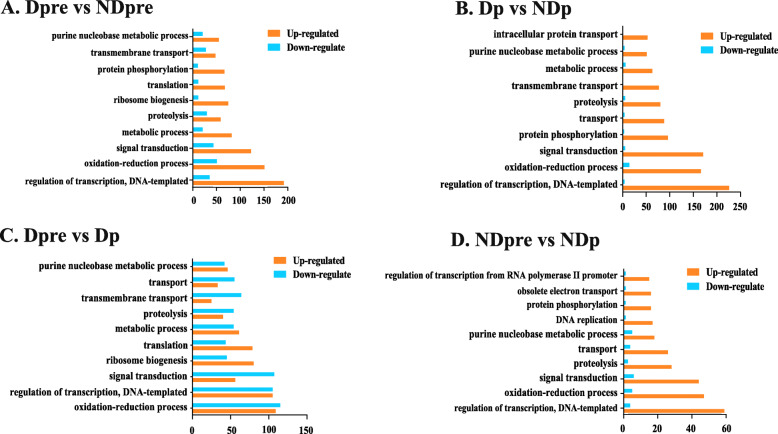
GO enrichment analysis. Ten GO items were selected according to the gene number of the four comparisons. The value of the horizontal ordinate represents the number of DEGs in each GO item. Up- or down-regulated genes are coded by different colors. **a** Ten GO items according to the gene number of the comparison of Dpre vs NDpre. **b** Ten GO items according to the gene number of the comparison of Dp vs NDp. **c** Ten GO items according to the gene number of the comparison of Dpre vs Dp. **d** Ten GO items according to the gene number of the comparison of NDpre vs NDp

To determine the potential function of identified DEGs, GO enrichment was performed. In all combinations, except for Dpre vs Dp, more genes were up-regulated. However, when we compared Dpre to Dp, there was little difference in the number of up- and down-regulated genes. Furthermore, more DEGs were assigned to the same category among different groups. Regulation of transcription, DNA-templated process, oxidation-reduction process, and signal transduction process were the top three in these four groups. The number of DEGs involved in ribosome biogenesis was much higher during diapause development than during normal development (Fig. 4). The subsequent analyses are based directly upon these results.

### Comparative analysis of genes involved in diapause

Based on the results of GO enrichment, we focused on the genes enriched in the oxidation-reduction process, regulation of transcription, DNA-templated process and signal transduction process, which were processes enriched in most DEGs. In addition, we also examined ribosome biogenesis.

**Fig. 5:**
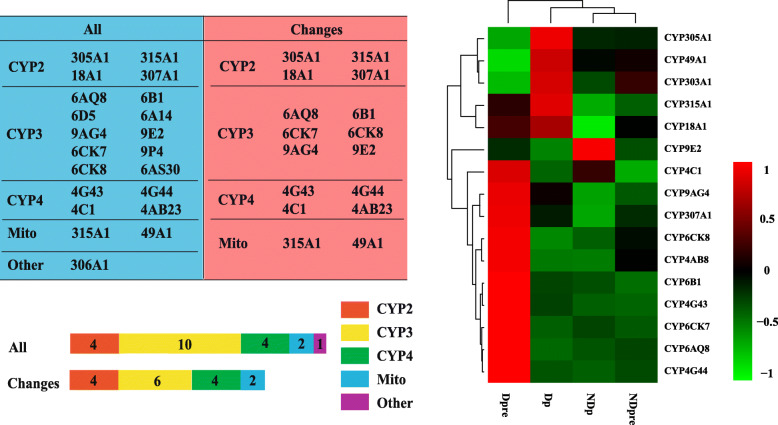
Cytochrome P450s (CYPs) genes selected in the *T. dendrolimi* transcriptome. The left table compares the number of CYPs between two groups. ALL means all the unigenes of the *T. dendrolimi* transcriptome, and Changes means the differentially expressed genes. The right heatmap graph shows the expression of differentially expressed CYPs genes

A total of 342 genes were identified in the oxidation-reduction process, and 16 of these belong to cytochrome P450s (CYP450s). In the *T. dendrolimi* transcriptome, 22 CYP450s were identified, and 16 were differentially expressed. These 16 genes belonged to 4 clans. In diapause stages (Dpre), 10 genes were up-regulated. In the pupae after diapause (Dp), five genes were highly expressed. Only one gene (CYP9E2) was highly up-regulated in normal pupae (NDp) (Fig. 5). These results show that the number of up-regulated genes during diapause was significantly higher than that in other stages.

There were 36 transcription factors differentially expressed during diapause development, and it appears that three kinds of transcription factors might be associated with diapause in *T. dendrolimi*.

**Fig. 6:**
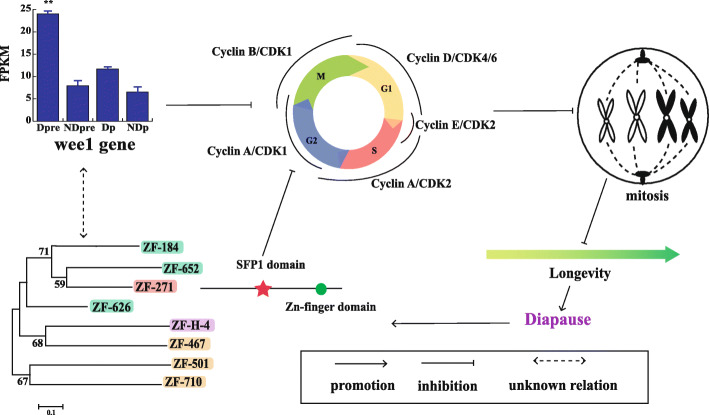
Speculated pattern diagram of zinc finger protein genes in the cell cycle. Eight zinc finger protein genes were screened in this study. ZF-271, indicated with a red background color, showed an interesting SFP1 domain, which is a putative transcriptional repressor regulating G2/M transition. The expression of the wee1 gene is also shown in the histogram. The *wee1* gene in other species, has the ability to let the cell remain at the G2 stage

The first kind is zinc finger protein. Three genes encoded zinc finger protein. Zinc finger protein 271, zinc finger 184, and zinc finger 544, were identified in the transcriptome. They were all up-regulated such that the expressions of these three genes in Dpre were higher than expression in Dp. Zinc finger protein gene 271 had an SFP domain. Genes containing this domain are putative transcriptional repressors during the G2/M (second gap period to mitotic period) transition. The *wee1* gene, encoding an inhibitory kinase, was up-regulated during diapause in *N. vitripennis* [36]. We obtained similar results in *T. dendrolimi* (Fig. 6). In addition, zinc finger protein 184 contained a GDT1 domain, which is a putative Ca^2+^/H^+^ antiporter. Ca^2+^/H^+^ antiporter, which maintains homeostasis, has been studied in plants, but there are few studies.

The second type of transcription factor is homeobox domain protein. In the transcriptome, 11 homeobox-containing genes were differentially expressed during diapause except for *pit1*, which was significantly up-regulated in the individuals that terminated diapause. Among these genes, homeobox protein homothorax (*hth*) had the greatest change in expression, followed by homeotic protein distal-less (*dll*) and homeobox protein six1 (*six1*). The gene expression of another homeobox protein *six1* was similar to *hth*; expression increased after entering diapause stage. And this gene may be involved in the regulation of cell proliferation, apoptosis, and embryonic development.

The third group of transcription factors is forkhead box protein. FOXOs have been identified as candidates for the molecular control of embryonic diapause in some species, like *Culex pipiens* [22, 37]. In *T. dendrolimi*, three forkhead box proteins (*foxo*), forkead box protein E3 (*foxe3*), and forkhead box protein D3 (*foxd3*) were identified. These genes were up-regulated both in diapause prepupae and resulting pupae, and they likely play a role in diapause development.

In the *T. dendrolimi* transcriptome, the expression of Protein phosphatase 2A (*PP2A*), which belonging to signal transduction process, was down-regulated during the diapause stage. This result was consistent with that obtained in the cotton bollworm (*Helicoverpa armigera*)*.* Low *PP2A* expression in diapause individuals contribute to the accumulation of *p-*Akt, and *p-*Akt leads to *H. armigera* diapause [24, 38].

**Fig. 7:**
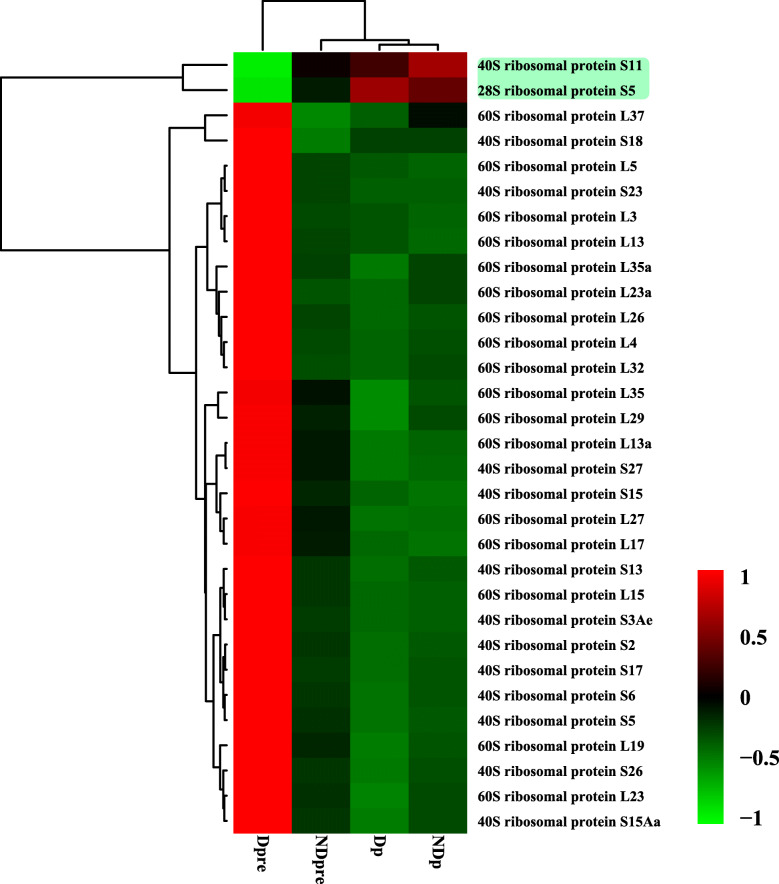
Hierarchical clustering analysis of ribosomal protein (RP) genes in *T. dendrolimi* at four different stages. FPKM values of 31 RP genes were used to construct the expression profiling. Hierarchical clustering of FPKM values was performed with the Pearson correlation-based metric and average linkage clustering method. The two genes with green background were down-regulated during the diapause development process

In addition to these three biological processes, ribosome biogenesis is also important in the control of cell growth and division in eukaryotes [39]. In this study, ribosome biogenesis involved 31 DEGs, and 29 genes were up-regulated during prepupal diapause. Only two genes, 40S ribosomal protein S11 (*rpS11*) and 28S ribosomal protein S5 (*rpS5*) were down-regulated during prepupal diapause. All of the 60S ribosomal proteins were up-regulated (Fig. 7).

Some genes, even those not involved in the biological process, with a considerable number of genes enriched, appear to be important in the diapause development of *T. dendrolimi*, such as *p53* and the DNA damage-regulated gene 1 (*pdrg1*), which gene expressions were significantly changed during diapause development. The transcriptional expressions of *Glutathione-S-transferase* (*GST*) and *UDP-glucuronosyltransferase* (*UDPGT*) were also up-regulated during *T. dendrolimi* prepupal diapause.

**Fig. 8:**
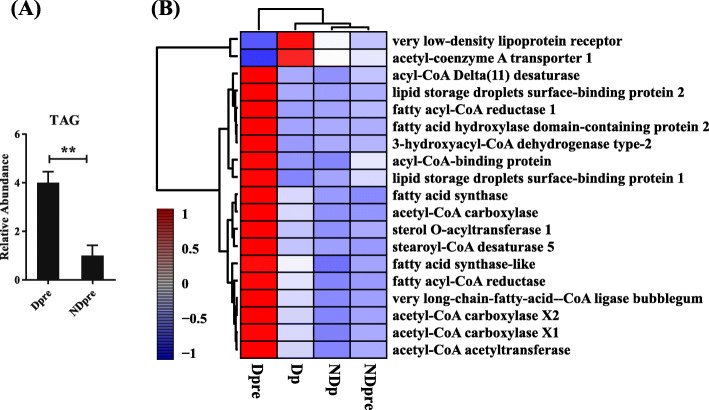
Lipid metabolism changes during diapause development. **a** TAG was measured in diapause and non-diapause *T. dendrolimi*. At least three independently collected samples for each treatment were analyzed. Dpre treatments were normalized to NDpre. The error bar represents the SEM; **p < 0.01. **b** Heatmap of expression patterns of lipid metabolism-related genes with fold changes > 1

Lipid metabolism is essential for energy homeostasis. Some diapausing insects use lipids for energy storage [40, 41]. During diapause, almost all selected lipid metabolism related genes were up-regulated, coinciding with the mobilization of TAG reserves (Fig. 8).

## Discussion

Diapause is a dynamic process accompanied by a series of physiological transitions. Several studies have focused on the general gene expression pattern of insect diapause without a clear elucidation of the diapause mechanism. This is due to the complexity of the diapause process as well as the variations among insect species. In this study, several genes were identified related to diapause in *T. dendrolimi*. Based on the results, 16 CYP450s gene differentially expressed at different stages in *T. dendrolimi*. CYP450s are hemoproteins involved in physiological processes such as biosynthesis of hormones and degradation of xenobiotics [42]. There are four clans in the P450 supergene family namely CYP2, CYP3, CYP4, and Mitochondrion (Mito) [43]. The CYP450 of *Schistosoma mansoni* was essential for worm survival and egg development [44]. CYP4G1 is related to cuticular hydrocarbon biosynthesis in *Drosophila* [45]. These genes were up-regulated in diapause individuals, suggesting that when *T. dendrolimi* entered diapause, the environmental conditions are unsuitable for survival. The conditions are worse than those under normal conditions. During this process, many harmful substances may be produced. A possible function for these genes is to reduce harmful substances, and maintain cellular homeostasis. This phenomenon still occurred in pupae after diapause (Dp) compared to pupae under normal condition (NDp). These data suggest that although diapause helps insects survive adverse environments, the process can also affect insect growth. Therefore, diapause development appears to be a metabolic tradeoff required for survival.

There were three kinds of transcription factors (zinc finger protein, homeobox domain protein, and forkhead box protein) differentially expressed during diapause development, which might be related to the diapause in *T. dendrolimi*. One of the most noticeable characteristics of diapause is the blockage of ontogeny, and this blockage always occurs with cell cycle cessation [46, 47]. In *N. vitripennis*, the S phase of the cell cycle disappeared in the beginning stage of diapause due to the cells being arrested in the G0/G1 (stop cell division to the first gap) and G2 phases [48]. Similarly, in the drosophilid fly, *Chymomyza costata*, the cell cycle of central nervous system (CNS) cells was arrested in the G0/G1 (86.6%) and G2 (12.8%) division phases during diapause [36]. In addition, the gene expression of *wee1* was upregulated both in *T. dendrolimi* and *N. vitripenni*. Therefore, the *wee1* gene might be a molecular marker of diapause in parasitoids.

During embryogenesis in *Tribolium castaneum*, *hth* involved in the segmentation process and is required for specification of body wall identities in the thorax [49]. In *D. melanogaster*, *hth* has different functions in different tissues. *Hth* located in the head leads to opposite effects on eye and antennal development as a negative regulator of eye development, and it acts with extradenticle (*exd*) to delimit the eye field and prevent inappropriate eye development [50]. Transcriptome factor *dll* plays a role in larval and adult appendage development [51]. In mammals, *six1* is essential for early neurogenesis in the development of olfactory epithelium [52]. They are important genes regulating myogenesis and highly up-regulated during diapause in *T. dendrolimi*. However, the aberrant expression of these genes may cause growth cessation. We infer that the genes that are up-regulated at diapause stage block the normal cell cycle in diapause *T. dendrolimi.*

In signal transduction, a variety of enzymes are speculated to play role in diapause. *PP2A* was an interesting candidate. It is a key serine-threonine protein phosphatase, which regulates several cellular processes, including metabolism, transcription, cell cycle, autophagy, and signal transduction [53, 54]. Cell cycle withdrawal, from G1 to S phase, is negatively related to PP2A activity [55, 56]. PP2A plays a multi-faceted role in the regulation of several pathways, such as the mTOR and the wnt signaling pathway, which are related to the cell cycle [53, 57]. PP2A may be involved in *T. dendrolimi* diapause.

In this study, we also focused the expression of genes associated with ribosome biogenesis. The rate of ribosome synthesis during diapause was lower than that of non-diapause eggs in *Bombyx mori*, so the up-regulation of ribosomal proteins can play an important role in blocking diapause [58, 59]. In *C. pipiens*, the expression of ribosomal protein S3a (*rpS3a*) was greatly reduced for a short time during the diapause stage. After the injection of *rpS3a* dsRNA into non-diapause females, follicle development was arrested, similar to diapause state [60]. Conversely, in *T. dendrolimi*, diapause prepupae had a higher expression of ribosomal proteins compared to non-diapause wasps. However, *B. mori* and *C. pipiens* enter diapause as adults, whereas *T. dendrolimi* enters diapause as prepupae. Although diapause decreased the level of metabolism, individuals may still require energy to maintain the diapause condition. This is the first report demonstrating that ribosomal protein is related to diapause in *T. dendrolimi*. The specific functions of the genes involved in diapause required additional study.

Some genes, even those not involved in the biological process, with a considerable number of genes enriched, appear to be important in the diapause development of *T. dendrolimi*. For examples, *p53* induces growth arrest or apoptosis and has a negative regulation effect on cell division by controlling an array of genes [61]. In addition, *p53* and *pdrg1* are involved in multiple cellular processes, such as apoptosis, DNA damage repair, and the cell cycle. In *Artemia sinica*, PDRG1 has an essential role in diapause termination and regulation of cell cycle during early embryonic development [62]. Blocking the expression of *pdrg1* in human colon cancer cells significantly reduced cell growth. Transcriptome-based analysis indicated that *p53* expression significantly increased during diapause, and contrasting results were observed for pdrg1 in *T. dendrolimi*. This result also revealed that apoptosis activity was enhanced, although the diapause individual remained in a dormant state. It is speculated that more harmful substances were accumulated during diapause. So, it was necessary to boost apoptosis activity for wasp to survive.

UDPGT is important in the elimination of toxic xenobiotics and endogenous compounds. GST belongs to a multifunctional protein family mainly located in the cytoplasm. Both are involved in cellular detoxification. The transcriptional expressions of *GST* and *UDPGT* were up-regulated during *T. dendrolimi* prepupal diapause. This was different from other species, such as the bee *Tetrapedia diversipes* and *Tetranychus urticae* in which transcripts were down-regulated during diapause [63]. In other insect species, the downregulation of *GST* and *UDPGT* might be correlated with a non-feeding condition, so the amount of exogenous substances can decrease accordingly. However, *T. dendrolimi* is an endoparasitoid wasp that spends its larval life within the host egg; starvation is a rare situation before the prepupae stage. Therefore, we believe that the up-regulation of these two genes in diapause *T. dendrolimi* might be due to increased resistance to unfavorable environmental conditions.

Genes related to lipid metabolism have important influence in the formation of triacylglycerol, which is the main caloric reserve during diapause. In addition, the expression of several genes significantly increased after diapause termination. This might be due to post-diapause development. Diapause *T. dendrolimi* can produce higher numbers of parasitized hosts than non-diapause *T. dendrolimi* [27]. The increased lipid storage could provide more energy for maintaining reproductive activities.

## Conclusions

Diapause is an important physiological process in insects, and it has potential as a method for the long-term storage of parasitoids. Using the characteristics of diapause, the developmental cycle can be increased to enhance the application efficiency of *T. dendrolimi*. This is important for the mass production of *Trichogramma* on a commercial scale. Although diapause has been successfully manipulated, the molecular mechanism of diapause remains largely unknown.

We compared the gene expression profiles among different diapause stages of *T. dendrolimi*. Our results were either consistent with previous studies or provided additional information useful for understanding the mechanism of diapause. Novel genes, such as *p53* and *pdrg1* might be relevant to diapause or apoptosis processes. Further studies are needed to elucidate the functions of candidate genes during diapause.

Our results provide crucial information that would be useful for generating a diapause genetics toolkit and establishing a method for improving the practical application of *T. dendrolimi* in biological control programs. However, diapause is a complex process, so additional studies will be required to elucidate the genetics of this insect adaptation.

## Methods

### Insect culture

Adult *T. dendrolimi* were collected from a corn field in Yitong, Jilin Province, China (125°11′E, 43°3′N) in 2015. Species identification was confirmed through the morphological characteristics of male genital capsules. A wasp population was maintained on the eggs of *Antheraea pernyi* (fresh eggs were dissected from the ovaries of female *A. pernyi* and were supplied in glass tubes to newly emerged *T. dendrolimi* for oviposition). The eggs were kept at 26 °C ± 1 °C, 60% ± 5% relative humidity (RH) with a 16:8 h (L:D) photoperiod.

### Diapause induction and termination

Approximately 1000 eggs of *A. pernyi* were parasitized for 2 h by approximately 2500 *T. dendrolimi* adults. The parasitized eggs were separated into two groups that received different treatments (Table 3). *T. dendrolimi* enteres diapause at the prepupa stage. Diapause prepupae and pupae after diapause were labeled Dpre and Dp, respectively. Non-diapause prepupae and non-diapause pupae were labeled NDpre and NDp. The methods for diapause induction and termination used in this study were based on those of Zhang et al. (2017) [18]. Specifically, diapause was induced by keeping *T. dendrolimi* at 12 °C and 60% ± 5% relative humidity (RH) with a 0:24 h (L:D) photoperiod for 30 d, and diapause was terminated by keeping *T. dendrolimi* at 3 °C and 60% ± 5% relative humidity (RH) with a 16: 8-h light: dark (L: D) photoperiod for 70 d (Fig. S1; Table 3). Then the individuals were kept at 26 °C ± 1 °C, 60% ± 5% relative humidity (RH) with a 16:8 h (L:D) photoperiod to develop.

**Table 3:** Different treatments for *T. dendrolimi* used in this study

Groups^**a**^	State	Treatment	Sampling Period	Sample Set
1	Non-diapause	26 ± 1 °C, 60 ± 5% RH, 16:8 L:D	Prepupal stage	NDpre
		26 ± 1 °C, 60 ± 5% RH, 16:8 L:D	Pupal stage	NDp
2	Diapause	12 ± 1 °C, 60 ± 5% RH, 0:24 L:D, 30 d	Prepupal stage	Dpre
		12 ± 1 °C, 60 ± 5% RH, 0:24 L:D, 30 d 3 ± 1 °C, 60 ± 5% RH, 0:24 L:D, 70 d 26 ± 1 °C, 60 ± 5% RH, 16:8 L:D, until pupal stage	Pupal stage	Dp

^a^ Groups 1 and 2 represent control and treatment, respectively

### RNA isolation and qualification and library preparation

Total RNA was extracted from four sample sets described above using TRIzol Reagent (Sigma Aldrich, St. Louis, MO, USA). Three replicates of four parasitized eggs were evaluated for each experiment. RNA degradation and contamination were monitored on 1% agarose gels. RNA purity, integrity, and concentration were checked using a NanoPhotometer® spectrophotometer (Implen, Westlake Village, CA, USA), RNA Nano 6000 Assay Kit of the Agilent Bioanalyzer 2100 system (Agilent Technologies, CA, USA), and Qubit® RNA Assay Kit in a Qubit® 2.0 Flurometer (Life Technologies, CA, USA), respectively. A total of 1.5 μg RNA per sample was used as input material for library preparation. Libraries used for sequencing were generated using the NEBNext® UltraTM RNA Library Prep Kit for Illumina® (NEB, USA) following manufacturer recommendations. Index codes were added to attribute sequences in each sample. Briefly, the mRNA was purified from the total RNA using poly-T oligo-attached magnetic beads. First-strand cDNA was synthesized using random hexamer primers and M-MuLV Reverse Transcriptase (RNase H-). Second-strand cDNA synthesis was subsequently performed using DNA Polymerase I and RNase H. After adenylation of 3′ ends of DNA fragments, a NEBNext Adaptor with hairpin loop structure was ligated to prepare for hybridization. To select cDNA fragments 250–300 bp in length, the library fragments were purified with an AMPure XP system (Beckman Coulter, Brea, CA, USA). Then, 3 μl USER Enzyme (NEB, USA) was used with size-selected, adaptor-ligated cDNA at 37 °C for 15 min followed by 5 min at 95 °C before PCR. Then PCR was performed with Phusion High-Fidelity DNA polymerase, Universal PCR primers, and Index (X) Primer. PCR products were purified (AMPure XP system, Beckman Coulter, USA) and library quality was assessed on the Agilent Bioanalyzer 2100 system. The prepared libraries were then sequenced with the Illumina HiSeq platform (Novogene, China), and paired-end reads were generated. The RNA-seq raw reads were deposited as project number PRJNA597631 in the Sequence Read Archive of the National Center for Biotechnology Information (NCBI). Three replicates were sequenced for each sample.

### De novo transcriptome assembly and functional annotation

Raw data (raw reads) were processed using in-house Perl scripts. Clean reads were obtained after removing adaptor, ploy-N, and low-quality reads. The Q20, Q30, GC-content, and sequence duplication level were measured. De novo transcriptome assembly was performed with Trinity 2.4.0 software ^[72]^ with min_kmer_cov set to 25 and all other parameters at the default values [64]. All assembled transcripts were aligned using DIAMOND v0.8.22, NCBI blast 2.2.28+, HMMER 3.0, and KAAS, with the protein and nucleotide sequences in five public databases (NR, NT, Pfam, KEGG, and Swiss-Prot) with a threshold of e < 0.00001. Functional annotation of all the transcripts was carried out using Blast2GO v2.5 [65]. The Gene Ontology (GO) and Clusters of Orthologs Groups for Eukaryotic Complete Genomes (KOG) databases were used. Then according to the results of annotation, the transcripts were filtered to remove those annotated to the same functional genes, which were identified as unigenes.

### Identification of differential expression genes (DEGs) and functional classification

Transcript expression levels were estimated by RSEM (RNA-Seq by Expectation-Maximization). A reference transcriptome was first generated by clustering all the samples unigenes i.e. Dpre, Dp, NDpre and NDp. Then the high-quality cleaned reads from each sample were aligned separately on a reference transcriptome using bowtie2 with default parameters to obtain the read count of each sample [66]. Considering the influence of sequencing depth and gene length of fragments counts, the read counts were transferred into FPKM (Fragments Per Kilobase of transcript per million mapped reads), according to the following formula to measure the expression level of each assembled transcript sequence. $\mathrm{FPKM}=\frac{\mathrm{total}\ \mathrm{exon}\ \mathrm{fragments}}{\mathrm{mapped}\ \mathrm{reads}\ \left(\mathrm{millions}\right)\times \mathrm{exon}\ \mathrm{length}\ \left(\mathrm{kb}\right)}.$ DEGs between four conditions (Dpre vs. NDpre; Dpre vs. Dp; NDpre vs. NDp; Dp vs NDp) were identified using the DESeq R package. Prior to differential expression analysis, the read counts in each sample were normalized using the default normalization method within the DESeq package. The resulting *P*-values were adjusted using the Benjamini-Hochberg method to control the false discovery rate (FDR). Transcripts with an adjusted *P*-value less than 0.05 and a |log2 (fold change) | greater than 1 found by DESeq were considered as differentially expressed. A Venn diagram (http://bioinformatics.psb.ugent.be/beg/) was used to compare the four DEG lists.

Gene Ontology (GO) enrichment analysis of DEGs was implemented by the GOseq R package [67]. For GO enrichment analysis, corrected *P*-values less than 0.05 were considered as significantly enriched in DEGs. The top 10 biological processes involved in these four lists were also analyzed. According to the results of GO enrichment analysis, candidate genes related to different stages during diapause development in *T. dendrolimi* were selected and analyzed.

### Validation experiment by quantitative real-time PCR (qPCR) analysis

Quantitative real-time PCR (qPCR) was performed on a qTOWER^3^G system (Analytikjena, Germany) using SYBR green PCR Master Mix (Tiangen, China). The thermal cycling conditions were as follows: one cycle of 95 °C for 5 min, 40 cycles of 95 °C for 5 s, and 60 °C for 15 s, following the melt curve program. The *GAPDH* of *T. dendrolimi* was selected as the internal standard to normalize the cDNA templates. Primers with product sizes of 100–200 bp were designed with Primer Premier 6 using the default settings. The selected candidate genes and corresponding primers are listed in Table 4. Three biological replicates were performed for each treatment with four technical replicates for each primer pair. The relative transcript expression levels among the different treatments were measured by the 2^-ΔΔCt^ method. Statistical analysis of the qPCR was conducted in SPSS 19.0 (SPSS Inc., Chicago, IL, USA). Data are presented as the mean ± standard error (SE). Significant differences between diapause and non-diapause were analyzed with independent sample *t-*tests.

**Table 4:** Candidate genes and primers used for qPCR analysis

Unigene ID	Gene name	Primer sequence	Amplicon size (bp)
Cluster-6290.39995	Heat shock protein 68 (*HSP68*)	F: TTCTGCCGATGAGACGCTTGG R: TGCCTTCACCGACACCGAGA	104
Cluster-6290.35917	Autophagy-related protein 2 (*ATG2*)	F: TCTGGAGCAGTAGGTGGAGTGT R: GCAGCCTCATGTCTGGCATCT	137
Cluster-6290.40838	Autophagy-related protein 9 (*ATG9*)	F: TCGGCATCGTCAACTTCGTCCT R: GCAGATAGAGGCGGCTGTAGGT	144
Cluster-6290.35139	Autophagy-related protein 13 (*ATG13*)	F: CGCAGCAGCAACAACAACAACC R: TGGTAGTGGTCGCCGATCTCTG	111
Cluster-6290.30161	*cyclin D*	F: GCCACGAGCTGATCGAGGAGAT R: GCTGTTGCTGTTGCTGCTGTTG	102
Cluster-6290.19679	Trehalose transporter (*Tret*)	F: CGAGGCGAACATCCAGAAGGT R: GGCAGCATCAGCATCGTCAC	111
Cluster-6290.35295	Trehalase (*Tre*)	F: GCCGACATCACAACCGAAGACA R: TCGTTCCAGAGCACCTCGTCAA	173
Cluster-6290.42522	Fatty acid synthase (*FAS*)	F: CGACGAGAAGCAGTTCAAGGC R: CGGACGAGAAGCAGACGAAGT	116
Cluster-6290.41101	lipid storage droplets surface-binding protein 2 (*LSD2*)	F: GGCCGTGTCGAGGATCAACTAC R: ACGAGCATCTCCAGGACGAAGG	162
Cluster-6290.38030	stearoyl-CoA desaturase 5 (*SCD5*)	F: TGGAGTCGCTGGAGGAATACAC R: ACGGGATCGGCATCGGTTTC	174
Internal control	Glyceraldehyde-3-phosphate dehydrogenase (*GAPDH*)	F: CCAGCCACCTACGACGAGATCA R: ACCACGAGATGAGCTTGACGAA	187

## Supplementary Information

**Additional file 1.**

## Data Availability

The datasets generated and/or analysed during the current study are available in the *Trichogramma dendrolimi* Transcriptome repository (BioProject accession number PRJNA597631.). Illumina sequence reads have been deposited at NCBI SRA database under the following accession numbers (Dpre: Sample: SAMN13678825, Reads: SRR13060941, SRR13060940, SRR13060937; NDpre: Sample: SAMN13679415, Reads: SRR13060936, SRR13060935, SRR13060934; Dp: Sample: SAMN13679232, SRR13060933, SRR13060932, SRR13060931; NDp: Sample: SAMN13679414, Reads: SRR13060930, SRR13060939, SRR13060938).

## Abbreviations

RNA-Seq: RNA sequencing
GO: Gene ontology
Dpre: Diapause prepupae
Dp: Pupae after diapause
NDpre: Non-diapause prepupae
NDp: Non-diapause pupae
NR: Non-redundant
DEGs: Differentially expressed genes
GAPDH: Glyceraldehyde phosphate dehydrogenase
*tre*: Trehalase
CYP450s: Cytochrome P450s
Mito: Mitochondrion
CNS: Central nervous system
*hth*: Homeobox protein homothorax
*dll*: Homeotic protein distal-less
*exd*: Extradenticle
*foxo*: Forkhead box protein O
*foxe3*: Forkhead box protein E3
*foxd3*: Forkhead box protein D3
PP2A: Protein phosphatase 2A
*rpS11*: Ribosomal protein S11
*rpS5*: Ribosomal protein S5
*rpS3a*: Ribosomal protein S3a
*pdrg1*: DNA damage-regulated gene 1
*UDPGT*: UDP-glucuronosyltransferase
*GST*: Glutathione-S-transferase
TAG: Triacylglycerol
KOG: Clusters of orthologs groups for eukaryotic complete genomes
FPKM: Fragments per kilobase million
RSEM: RNA-Seq by expectation-Maximization
FDR: False discovery rate
qPCR: Quantitative real-time PCR
