# Supplementary figures and images for: High-throughput profiling of diapause regulated genes from Trichogramma dendrolimi (Hymenoptera: Trichogrammatidae)
Source: BMC Genomics. 2020 Dec 4;21:864. doi: 10.1186/s12864-020-07285-4 (PMC7718664; doi:10.1186/s12864-020-07285-4)

## diapause density

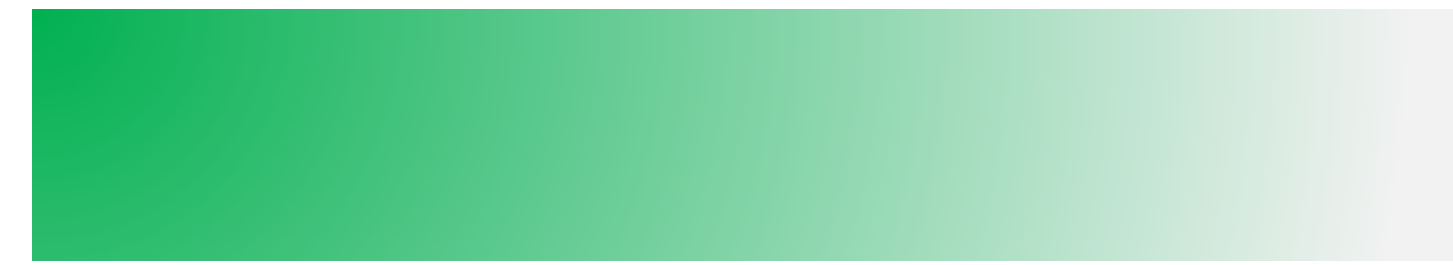

high

low

## Diapause development

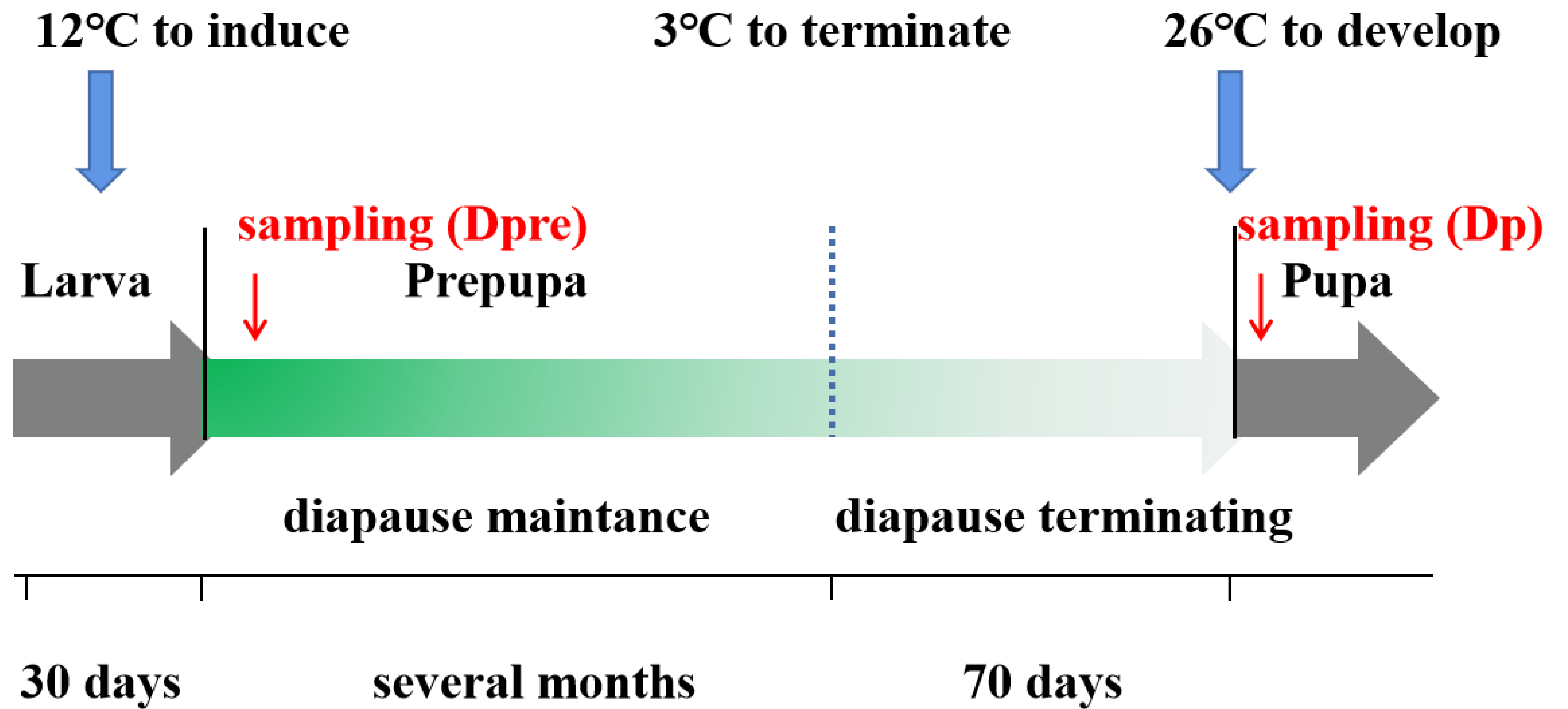

## Normal development

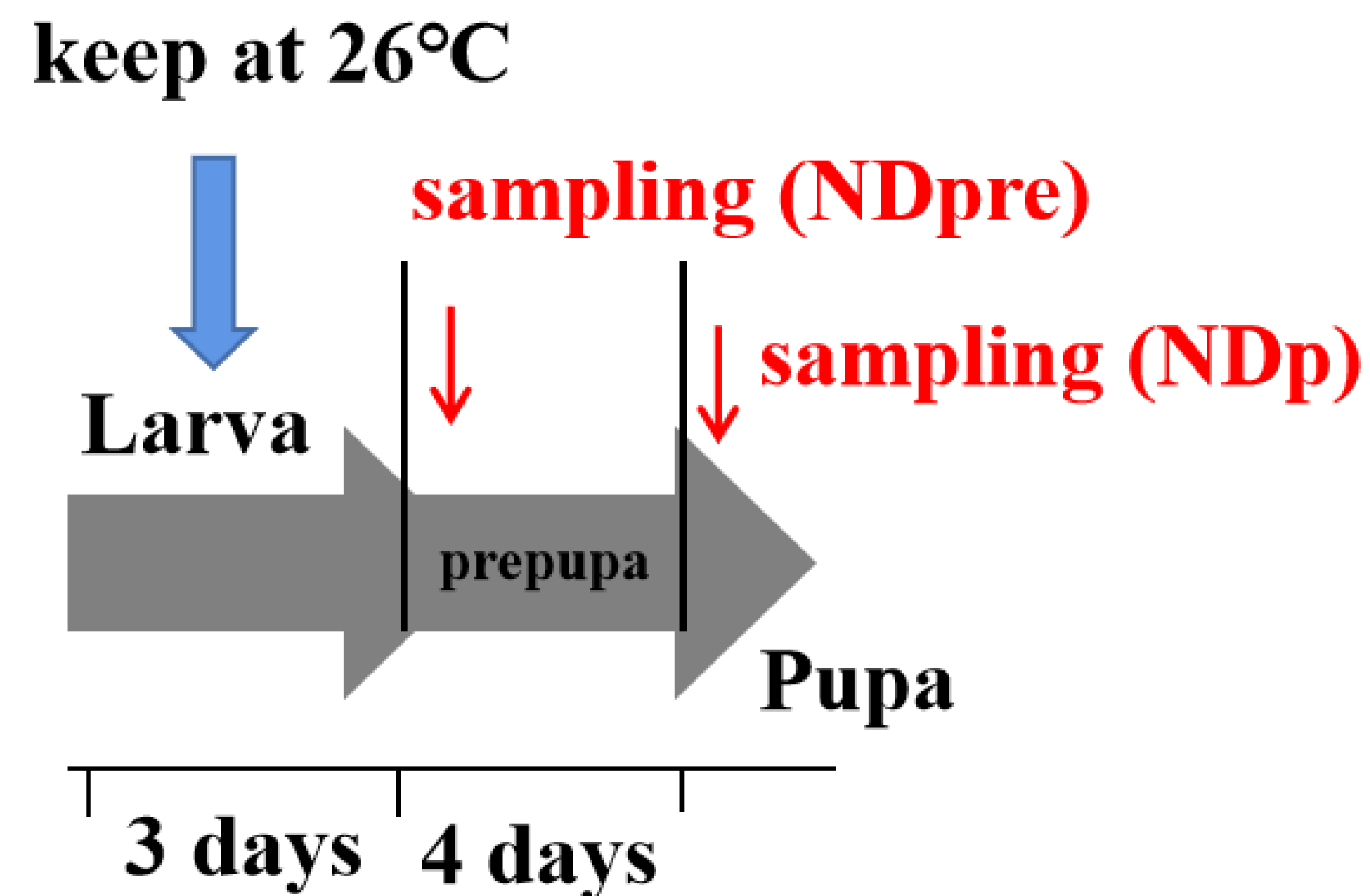

Supplement: Supplementary file 1 — Additional file 1. [file 12864_2020_7285_MOESM1_ESM.pdf]
